# The construction of a dual direct Z-scheme NiAl LDH/g-C_3_N_4_/Ag_3_PO_4_ nanocomposite for enhanced photocatalytic oxygen and hydrogen evolution†

**DOI:** 10.1039/d0na01074j

**Published:** 2021-02-26

**Authors:** S. Megala, P. Ravi, P. Maadeswaran, M. Navaneethan, M. Sathish, R. Ramesh

**Affiliations:** Department of Physics, Periyar University Salem-636011 Tamil Nadu India rameshphys@gmail.com; Electrochemical Power Sources Division, Central Electrochemical Research Institute Karaikudi-630006 Tamil Nadu India; Academy of Scientific and Innovative Research (AcSIR) Ghaziabad-201002 India; Department of Energy Science, Periyar University Salem-636011 Tamil Nadu India; Nanotechnology Research Center (NRC), Faculty of Engineering and Technology, SRM Institute of Science and Technology Kattankulathur Chennai 603203 Tamil Nadu India

## Abstract

Dual direct Z-scheme photocatalysts for overall water decomposition have demonstrated strong redox abilities and the efficient separation of photogenerated electron–hole pairs. Overall water splitting utilizing NiAl-LDH-based binary and ternary nanocomposites has been extensively investigated. The synthesized binary and ternary nanocomposites were characterized *via* XRD, FTIR, SEM, HRTEM, XPS, UV-DRS, and photoelectrochemical measurements. The surface wettability properties of the prepared nanocomposites were measured *via* contact angle measurements. The application of the NiAl-LDH/g-C_3_N_4_/Ag_3_PO_4_ ternary nanocomposite was investigated for photocatalytic overall water splitting under light irradiation. In this work, we found that in the presence of Ag_3_PO_4_, the evolution of H_2_ and O_2_ is high over LCN30, and 2.8- fold (O_2_) and 1.4-fold (H_2_) activity increases can be obtained compared with the use of LCN30 alone. It is proposed that Ag_3_PO_4_ is involved in the O_2_ evolution reaction during water oxidation and g-C_3_N_4_ is involved in overall water splitting. Our work not only reports overall water splitting using NiAl-LDH-based nanocomposites but it also provides experimental evidence for understanding the possible reaction process and the mechanism of photocatalytic water splitting. Photoelectrochemical measurements confirmed the better H_2_ and O_2_ evolution abilities of NiAl-LDH/g-C_3_N_4_/Ag_3_PO_4_ in comparison with NiAl LDH, g-C_3_N_4_, Ag_3_PO_4_, and LCN30. The observed improvement in the gas evolution properties of NiAl LDH in the presence of Ag_3_PO_4_ is due to the formation of a dual direct Z-scheme, which allows for the easier and faster separation of charge carriers. More importantly, the LCNAP5 heterostructure shows high levels of H_2_ and O_2_ evolution, which are significantly enhanced compared with LCN30 and pure NiAl LDH.

## Abstract 2

Dual direct Z-scheme photocatalysts for overall water decomposition have demonstrated strong redox abilities and the efficient separation of photogenerated electron–hole pairs.

## Graphical abstract

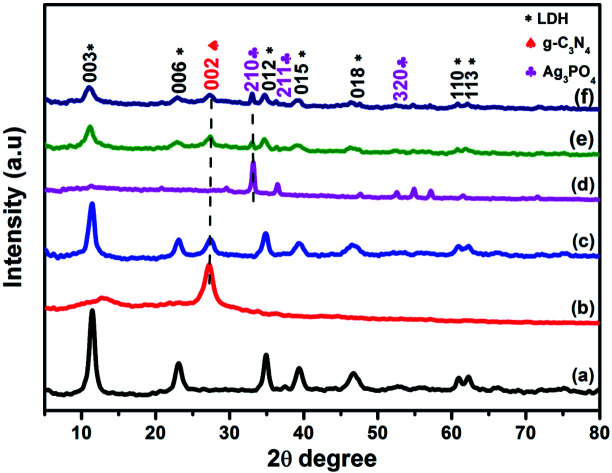

## 1. Introduction

The photocatalytic production of hydrogen and oxygen is one of the most promising approaches for overcoming both the energy and environmental issues that currently exist worldwide. This is because it is the best avenue for the direct generation of energy carriers that can be easily stored and transformed into electric energy.^1,2^ The water-splitting process consists of two half-reactions: water reduction with the evolution of H_2_; and water oxidation to produce O_2_.^3^ Oxygen evolution is hard to realize compared to hydrogen evolution due to the higher energy barrier. Moreover, 1 mol of water decomposes into 1 mol of hydrogen and 1/2 mol of oxygen, and 237 kJ is theoretically required for the water-splitting reaction under standard conditions. In general, a photocatalyst with a low (more positive) conduction band bottom or a high (more negative) valence band potential reduces the driving forces of photocatalytic reduction and oxidation reactions.^4^ Obtaining semiconductor photocatalysts with suitable redox potentials for water splitting is very difficult. At present, Z-scheme photocatalysts have generated much attention for achieving the complete water-splitting reaction. They can simultaneously reduce the recombination of charge carriers and retain strong redox abilities in the form of photo-excited electron–hole pairs.^5^

To date, 2D layered materials have also attracted extensive attention for photocatalytic water splitting. As a representative of layered materials, layered doubled hydroxides (LDHs) have attracted much attention in the field of catalysis due to their structures. Also, they have large surface-to-volume ratios, excellent chemical stability, low cost, low toxicity, rich redox activity, and appropriate band edges.^6,7^ LDHs are also known as anionic clays or hydrotalcite-like compounds, and they can be represented by the general formula [M^2+^_1−*x*_M^3+^_*x*_(OH)_2_]^*x*+^[A_*x*/*n*_]^*n*−^·*m*H_2_O, where M^2+^ and M^3+^ represent divalent and trivalent metal cations, respectively, and A^*n*−^ represents interlayer anions.^8^ Unfortunately, single photocatalysts show limited performance during simultaneous water oxidation and reduction, and there is a need to develop heterojunction photocatalysts to enhance the redox reaction.^9,10^ In the available literature there are many examples of LDHs in suitable redox composites, *e.g.*, with g-C_3_N_4_,^11–13^ CeO_2_,^8^ rGO,^7^ Cu_2_O,^14^ CdS,^15^ Ni_5_P_4_,^16^ CoP,^17^ and carbon black,^18^ and the water-splitting half-reaction (water reduction and oxidation) activities have been significantly increased. Hence, if suitable water redox composites are formed with LDHs, gaseous hydrogen and oxygen evolution could be achieved through water splitting.^18^ Polymeric g-C_3_N_4_ has recently attracted attention due to its enhanced visible response, tunable band structure, and extraordinary thermal and chemical stability.^19,20^ Formerly, our group studied the photocatalytic H_2_ evolution rates of LDH/g-C_3_N_4_ (in the presence of TEOA) composites through water splitting, which exhibited remarkable activities.^11^ Moreover, the excellent photocatalytic performance of LCN30 could be further improved through increasing the lifetimes of electrons and holes. Therefore, constructing an LCN30 ternary composite could not only facilitate charge carrier transfer at the interface of LDH and g-C_3_N_4_ but it could also improve the harvesting abilities for visible light.^21,22^

Several heterostructures have been fabricated to improve water splitting efficiencies in the literature.^23–25^ It is worth noting that silver orthophosphate (Ag_3_PO_4_) is a potential candidate in the field of photocatalytic water splitting due to its superior quantum efficiency under light irradiation.^26^ Loading Ag_3_PO_4_ onto P–g-C_3_N_4_ increased the photocatalytic hydrogen evolution activity 1.2-fold compared with pure P–g-C_3_N_4_.^27^ Tian *et al.* reported that Ag_3_PO_4_ coupled with g-C_3_N_4_/MOS_2_ to form a Z-scheme configuration can improve oxygen evolution during water splitting.^28^ The hybridization of Ag_3_PO_4_ with other semiconductors has resulted in superior water splitting performance. An artificial Z-scheme photocatalytic system is a gift for overall water splitting.^29^ This is because the Z-scheme system exhibits high quantum yields at a given wavelength and operates over a wide range of the solar spectrum.^30^ Z-scheme-type ternary photocatalytic composite materials have been proven to be more efficient than binary composite photocatalysts with single Z-scheme channels. The recombination of photoinduced electron–hole pairs can be suppressed due to an increased number of transfer channels. Moreover, the traditional charge-transfer process is not favorable for oxidation–reduction reactions.^31–33^ Herein, we designed a dual direct Z-scheme photocatalyst, in which NiAl-LDH/g-C_3_N_4_/Ag_3_PO_4_ serves as the H_2_ and O_2_ evolution photocatalyst. The synthesized dual direct Z-scheme photocatalyst shows enhanced photocatalytic activity for overall water splitting under light irradiation. Lastly, the enhanced activity of the dual direct Z-scheme photocatalyst can be attributed to Ag_3_PO_4_.

## 2. Experimental section

### 2.1. Materials

Nickel nitrate hexahydrate, aluminum nitrate nonahydrate, urea, disodium hydrogen phosphate dihydrate, and methylene blue stain were purchased from Merck India. Melamine was purchased from Loba Chemie Pvt. Ltd. Silver nitrate was purchased from Himedia chemical industry. All reagents were of analytical grade and used without further purification. DI water was used throughout the experiments.

### 2.2. Synthesis of the NiAl-LDH/g-C_3_N_4_ composite (LCN)

Pristine g-C_3_N_4_ was prepared *via* the heat treatment of melamine, as reported previously.^11^ To obtain NiAl-LDH/g-C_3_N_4_, as prepared g-C_3_N_4_ sheets (30 wt%) were dispersed in 25 ml of DI water through 20 min of sonication. In the meantime, an aqueous solution (60 ml) of Ni(NO_3_)_2_·6H_2_O and Al(NO_3_)_3_·9H_2_O was prepared at a 2 : 1 ratio. This clear green solution was added dropwise into 25 ml of the above g-C_3_N_4_ dispersion and vigorous stirring was carried out for 1 h. Then, 0.4 M urea was added into the suspension at room temperature. After being stirred for another 30 min, the NiAl-LDH/g-C_3_N_4_ suspension was transferred into a 100 ml Teflon-lined stainless-steel autoclave and heated at 140 °C for 10 h. The precipitate was collected, washed, and dried at 80 °C for 24 h. The obtained NiAl-LDH/g-C_3_N_4_ composite was denoted as LCN30.

### 2.3. Synthesis of NiAl-LDH/g-C_3_N_4_/Ag_3_PO_4_ (LCNAP)

**Scheme 1:**
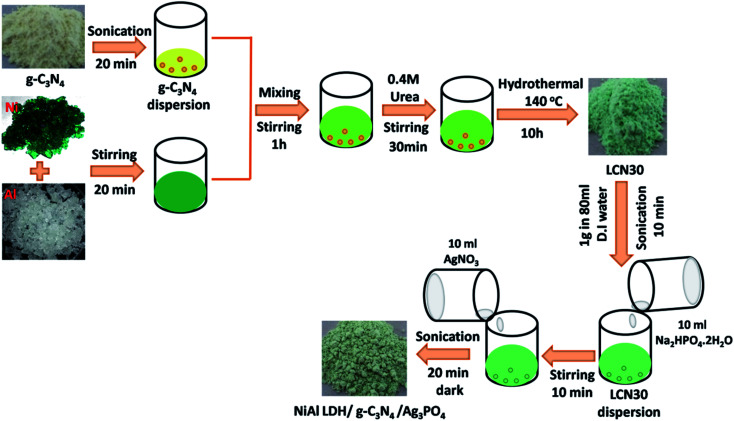
The fabrication process of the NiAl-LDH/g-C_3_N_4_/Ag_3_PO_4_ composite.

1 g of NiAl-LDH/g-C_3_N_4_ (30 wt%) was dispersed in 80 ml of DI water *via* ultrasonication for 10 min. Then, 10 ml of Na_2_HPO_4_·2H_2_O solution was added into the above dispersion, followed by stirring for 10 min. Subsequently, 10 ml of AgNO_3_ solution was dropped into the above suspension, and this was then stirred for 15 min. After that, the resulting nanocomposite suspension was sonicated for 20 min in the dark at room temperature. The obtained final product was filtered and washed (with DI water and ethanol) several times and dried at 60 °C for 24 h. Nanocomposites were prepared *via* varying the ratio between Ag_3_PO_4_ and NiAl-LDH/g-C_3_N_4_, and these were designated as NiAl-LDH/g-C_3_N_4_/AgPO_*x*_ (*x* = 5 and 10 wt%) and labeled as LCNAP5 and LCNAP10, respectively. The fabrication process for the NiAl-LDH/g-C_3_N_4_/Ag_3_PO_4_ composite is described in Scheme 1.

### 2.4. Characterization

The crystal structures and phase compositions of the synthesized products were identified in the range of 5 to 80° using a Rigaku Miniflex (Cu Kα Radiation) XRD diffractometer (operating at 40 kV and 30 mA). The surface functional groups of the materials were analyzed using a Brucker Tensor 27 Fourier-transform infrared spectrometer. The photoluminescence (PL) spectra were obtained using a JASCO-FP-8200 fluorescence spectrometer with an excitation wavelength of 380 nm. UV-visible diffuse reflectance spectra were obtained using a PerkinElmer Lambda 25 diffuse reflection spectrometer. X-ray photoelectron spectra characterization was carried out using an ESC 3400 photoelectron spectroscope system. FE-SEM and HR-TEM images were obtained using JEOL JSM 7001F and JEOL JEM 2100F electron microscopes. An Oxford EDS system was used to obtain EDS spectra and elemental maps. The water contact angles of the materials were measured with a Holmarch contact angle meter. A conventional three-electrode system using a BioLogic Science electrochemical workstation (France) was used to determine the photoelectrochemical properties of the samples. 20 mg of prepared catalyst and 100 μL of Nafion were ultrasonicated in 500 μL of ethanol for 30 min to get a slurry, which was then evenly spin-coated onto cleaned FTO (1 cm^2^) conducting glass to serve as the working electrode. Ag/AgCl (in saturated KCl solution) acted as the reference electrode and Pt acted as the counter electrode, and experiments were performed in 0.5 M Na_2_SO_4_ solution. A 150 W xenon lamp with a 1.5 AM filter was used for light irradiation for photoelectrochemical measurements. Transient photocurrent (I-t) measurements were performed for 500 s according to the chronoamperometry technique. Nyquist plots were obtained in the range between 1 MHz and 0.1 Hz *via* the electrochemical impedance spectroscopy (EIS) technique with an AC amplitude of 10 mV at the open circuit potential. Mott–Schottky (M–S) studies were done at a frequency of 1 kHz under dark conditions.

### 2.5. Measurements of the photocatalytic activity

The photocatalytic efficiencies of the prepared photocatalysts were tested based on the water-splitting reaction in a sealed air-tight quartz reactor under simulated light irradiation. The reactor contained 50 mg of photocatalyst in 50 ml of distilled water with a scavenger. For the H_2_ evolution reaction, 10% methanol (hole scavenger) solution [45 ml water + 5 ml methanol] was used, whereas 0.05 M AgNO_3_ (electron scavenger) solution was used for the O_2_ evolution reaction. The solution was stirred to prevent coagulation and subjected to light irradiation using a 250 W quartz tungsten halogen lamp as a light source. Before the photocatalytic reaction, the solution was purged with N_2_ gas for 1 h to remove dissolved gases. During the reaction, H_2_ and O_2_ gas samples were collected and analyzed using offline gas chromatography (CG-14C, Shimadzu, Japan) equipped with a TCD detector and 5 Å molecular sieve column with nitrogen as the carrier gas. Apparent quantum yields were determined *via* the following equations:

For H_2_ evolution:
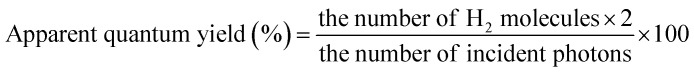


For O_2_ evolution:
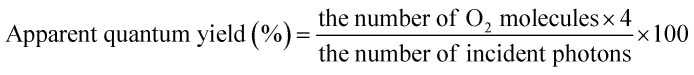


## 3. Results and discussion

### 3.1. Phase composition and morphology analyses

**Fig. 1:**
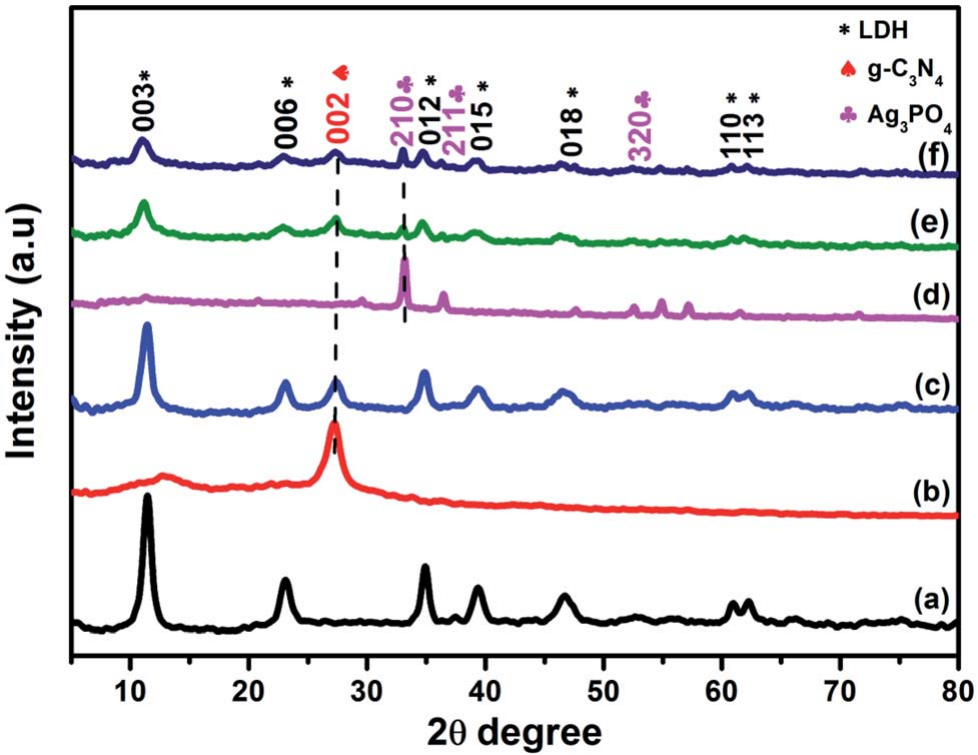
XRD patterns of (a) NiAl-LDH, (b) g-C_3_N_4_, (c) LCN30, (d) Ag_3_PO_4_, and (e and f) LCNA (5 and 10 wt%) nanocomposites.

Fig. 1 presents the X-ray diffraction patterns of pure NiAl-LDH, g-C_3_N_4_, Ag_3_PO_4_, and LCNAP composites with different Ag_3_PO_4_ wt% values. From Fig. 1a, the diffraction peaks at 11.54°, 23.14°, 34.86°, 39.36°, 46.7°, 60.9°, and 62.3° match well with the (003), (006), (012), (015), (018), (110), and (113) planes, respectively, of pure NiAl-LDH (JCPDS card no. 15-0087).^34,35^ The diffraction peak at 27.1° is assigned to the (002) plane of graphitic carbon nitrate (JCPDS card no. 41-1487) (Fig. 1c).^36^ In LCNAP (Fig. 1e and f), the peaks at 33.18°, 36.53°, and 54.96° could be ascribed to the (210), (211), and (320) planes of the cubic phase of Ag_3_PO_4_, and this clearly showed the presence of both g-C_3_N_4_ and Ag_3_PO_4_ in the composites (JCPDS card no. 06-0505). Loading with Ag_3_PO_4_ causes a decrease in the peak intensity of the (003) plane of LCN30, as it steadily covers the surface of LCN30, controlling the growth of that peak. The XRD pattern confirms the presence of multiple phases (g-C_3_N_4_ and Ag_3_PO_4_) in the LCNAP composite.

**Fig. 2:**
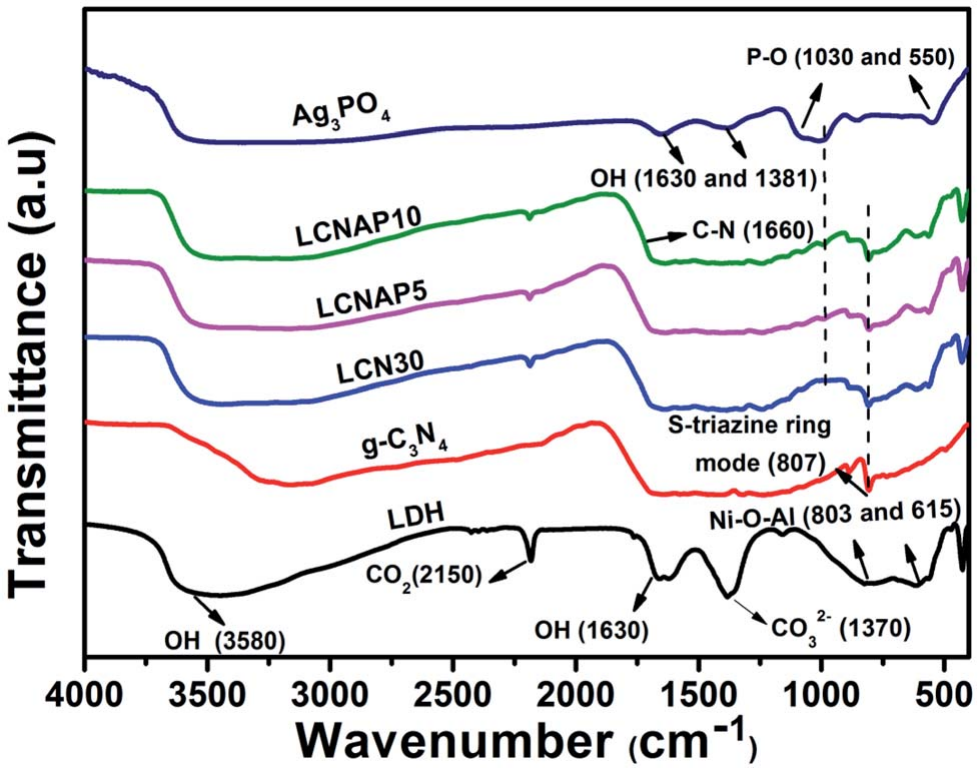
FTIR spectra of the NiAl-LDH, g-C_3_N_4_, LCN30, Ag_3_PO_4_, and LCNAP nanocomposites.

The chemical compositions and vibrational modes of the synthesized samples are shown in Fig. 2. The broad bands at around 3580, 1630, and 1381 cm^−1^ correspond to the stretching and bending vibrations of interlayer and surface (O–H) water molecules. The band at 1370 cm^−1^ indicates the stretching vibrations of interlayer anionic carbonate groups. The band at 2150 cm^−1^ shows the presence of CO_2_ in the NiAl-LDH sample.^37,38^ Additionally, the peaks below 800 cm^−1^ represent the M–O (Ni–O and Al–O) and M–O–M (Ni–O–Al) bond stretching of NiAl-LDH.^39^ g-C_3_N_4_ exhibits a characteristic peak at 3200 cm^−1^ corresponding to the N–H stretching vibration mode, whereas the peak appearing at 1660 cm^−1^ is related to the aromatic C=N stretching vibration. The peak at 806 cm^−1^ is related to the stretching mode of the *s*-triazine unit of g-C_3_N_4_.^40,41^ In the case of pure Ag_3_PO_4_, the peaks observed at 1030 and 550 cm^−1^ can be ascribed to the P–O stretching vibration of PO_4_.^42^ As can be seen, all the characteristic peaks of NiAl-LDH, Ag_3_PO_4_, and g-C_3_N_4_ are observed in the LCNAP samples, confirming the formation of NiAl-LDH/g-C_3_N_4_/Ag_3_PO_4_ nanocomposites.

**Fig. 3:**
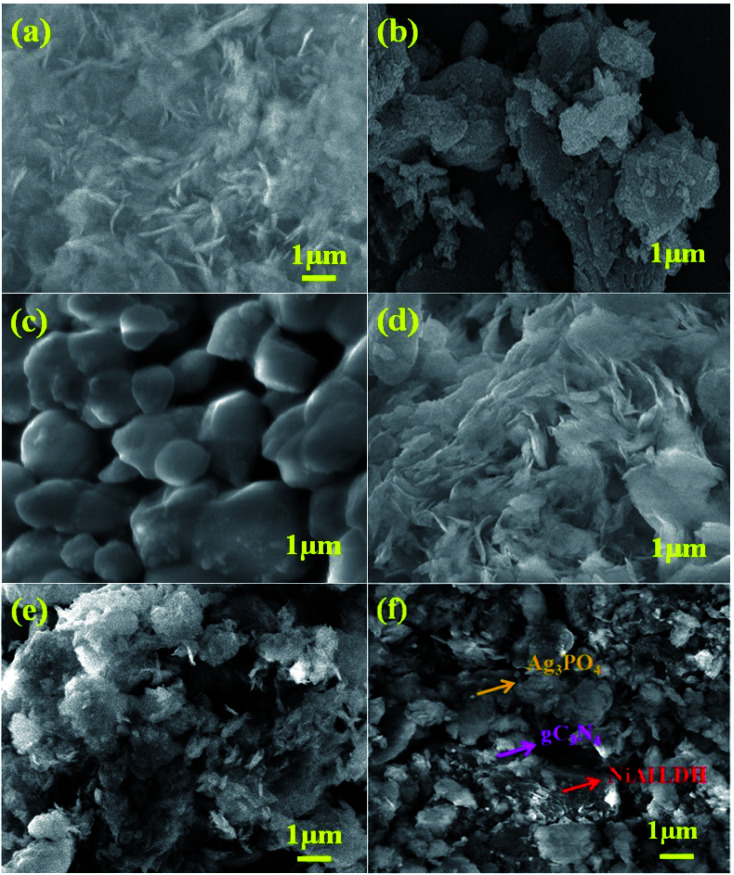
FE-SEM images of (a) NiAl-LDH, (b) g-C_3_N_4_, (c) Ag_3_PO_4_, and the (d) LCN30, (e) LCNAP5, and (f) LCNAP10 nanocomposites.

**Fig. 4:**
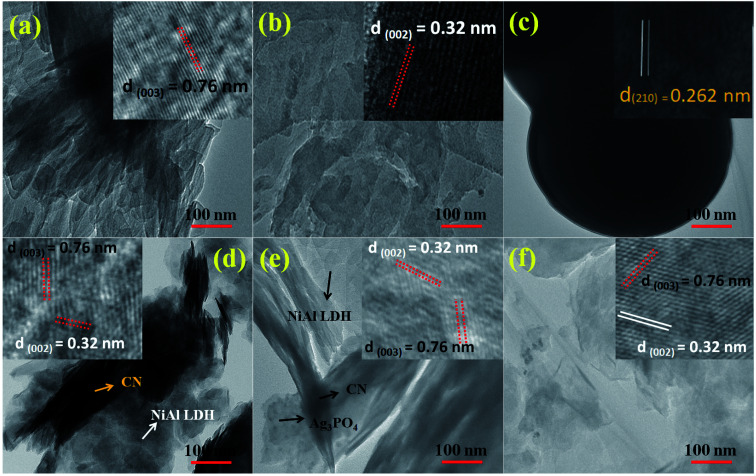
TEM and HRTEM images of (a) NiAl-LDH, (b) g-C_3_N_4_, (c) Ag_3_PO_4_, and the (d) LCN30, (e) LCNAP5, and (f) LCNAP10 nanocomposites.

**Fig. 5:**
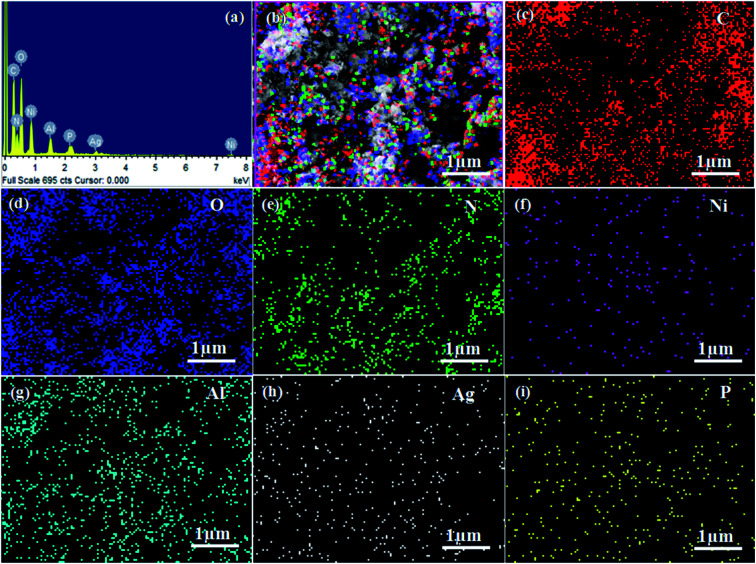
(a) The energy-dispersive X-ray (EDX) spectrum and (b–i) elemental mapping (EDS) images of the LCNAP5 nanocomposite.

The surface morphologies and microstructures of the as-fabricated samples were probed *via* FE-SEM, TEM, and HR-TEM techniques. Fig. 3(a and b) and 4(a and b) show pure LDH and g-C_3_N_4_, demonstrating their sheet structures. From the SEM image, it can be observed that pure Ag_3_PO_4_ exhibited irregular spheroidal structures with sizes ranging from 200–400 nm, as depicted in Fig. 3c. The synthesis of Ag_3_PO_4_ was carried out based on silver–ligand complexes with Na_2_HPO_4_ at room temperature. The irregular structure of Ag_3_PO_4_ was due to the direct reaction of AgNO_3_ and Na_2_HPO_4_ in water.^43^Fig. 3f shows an SEM image of LCNAP10; both the g-C_3_N_4_ sheets and Ag_3_PO_4_ nanoparticles appear to be uniformly distributed on the surfaces of the NiAl-LDH layers. After the formation of the ternary composite, this helps to create better photoexcited electron–hole separation, hence improving the photo-oxidation and -reduction compared with LCN30. To further establish the emergence of g-C_3_N_4_ and Ag_3_PO_4_ on the surface of NiAl-LDH, TEM analysis of LCNAP5 was carried out (Fig. 4). Fig. 4e reveals that the heterostructure possesses multiple overlapping layers, with g-C_3_N_4_ and Ag_3_PO_4_ electrostatically assembled on the surfaces of the NiAl-LDH sheets, resulting in the formation of the LCNAP nanocomposite. However, the Ag_3_PO_4_ nanoparticles are not easily detected in the LCNAP5 composite (Fig. 3e and 4e) because they are loaded at a very low wt% on the surface of LCN30. Furthermore, HRTEM analysis was used to provide pivotal evidence for the suitable interaction of components and the formation of the LCNAP5 heterostructure. As shown in the insets of Fig. 4a–c, the lattice fringe patterns of NiAl-LDH, g-C_3_N_4_, and Ag_3_PO_4_ have d-spacing values of 0.76, 0.32, and 0.262 nm, respectively, corresponding to the (003), (002), and (210) planes, respectively, which are consistent with their XRD patterns. In addition, the lattice fringes of Ag_3_PO_4_ are not visible in the composite samples (Fig. 4e and f), revealing the existence of heavy-atom formation. The energy-dispersive X-ray spectrum and elemental mapping of the LCNAP5 nanocomposite are shown in Fig. 5a–i, confirming the existence of C, O, N, Ni, Al, Ag, and P elements. These results indicate the successful formation of the NiAl-LDH/g-C_3_N_4_/Ag_3_PO_4_ nanocomposite.

### 3.2. Surface chemical composition

**Fig. 6:**
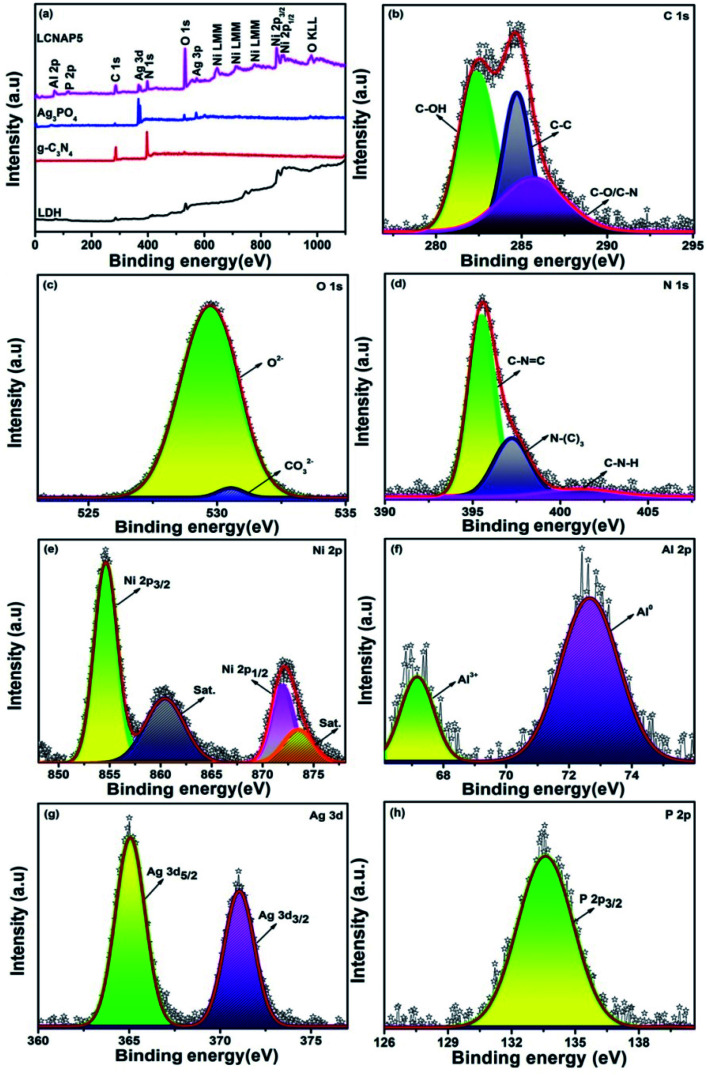
(a) XPS survey spectra of pure LDH, g-C_3_N_4_, Ag_3_PO_4_, and the LCNAP5 nanocomposite, and high-resolution (b) C 1s, (c) O 1s, (d) N 1s, (e) Ni 2p, (f) Al 2p, (g) Ag 3d, and (h) P 2p spectra of the LCNAP5 nanocomposite.

The chemical and electronic structures of the synthesized LCNAP5 nanocomposite were investigated *via* X-ray photoelectron spectroscopy (XPS) studies. The survey spectrum in Fig. 6a displays the existence of Ag, P, Ni, Al, C, N, and O elements. The high-resolution spectra of pure g-C_3_N_4_ and Ag_3_PO_4_ are presented in Fig. S1 and S2.† Characteristic C 1s peaks in the spectrum were observed at 282.3, 284.7, and 285.6 eV, relating to the C–OH, C–C, and C–O/C–N bonds of the prepared samples; these also indicated the presence of intercalated carbonate anions in the layer structure of NiAl-LDH.^8,44,45^ Peaks at 529 and 530.5 eV were also present in the O 1s spectrum: the first peak indicated lattice oxygen (O^2−^) in the pure and heterogeneous multiphase nanocomposite; and the second peak was attributed to oxygen bonds in the carbonate anions in the LDH interlayers (Fig. 6c). The peak at 531.5 eV indicated the presence of C–OH bonds in pure Ag_3_PO_4_ (Fig. S2†).^45,46^ In the N 1s spectrum (Fig. S1† and 6d), the three peaks located at 395.2, 396.4, and 401.1 eV in the pure g-C_3_N_4_ spectrum and at 395.4, 397.1, and 401.3 eV in the LCNAP5 spectrum could be assigned to the nitrogen-bonded species of C–N=C, N–(C)_3_, and C–N–H, respectively.^47^ In the Ni 2p spectrum, the 2p_3/2_ and 2p_½_ peaks at 854.6 and 871.8 eV, respectively, indicated the existence of Ni^2+^, whereas the satellite peaks appearing at 860.3 and 873.4 eV signified the presence of high-spin divalent Ni^2+^ in this hydrotalcite sample (Fig. 6e).^48^ Moreover, in the Al 2p spectrum (Fig. 6f), trivalent Al^3+^ can be determined with a binding energy of 67 eV, and a binding energy at 72 eV indicates Al(0).^49,50^ The observation of Ag and P peaks manifested the presence of Ag_3_PO_4_ in the composite, as shown in Fig. 6g and h. The spectra in Fig. 6g and S2† show that the characteristic peaks of Ag 3d were located at 367.65 and 373.72 eV for pure Ag_3_PO_4_ and at 365.03 and 371.02 eV for LCNAP5. Compared with pure Ag_3_PO_4_, the binding energies of the LCNAP5 composite were decreased, which indicates strong interfacial interactions between Ag_3_PO_4_ and LCN30.^51^ From Fig. 6h and S2,† the peak observed at 133.57 eV indicates the presence of phosphate ions in the P 2p orbital state.^52^

### 3.3. Optical properties and energy band structure

**Fig. 7:**
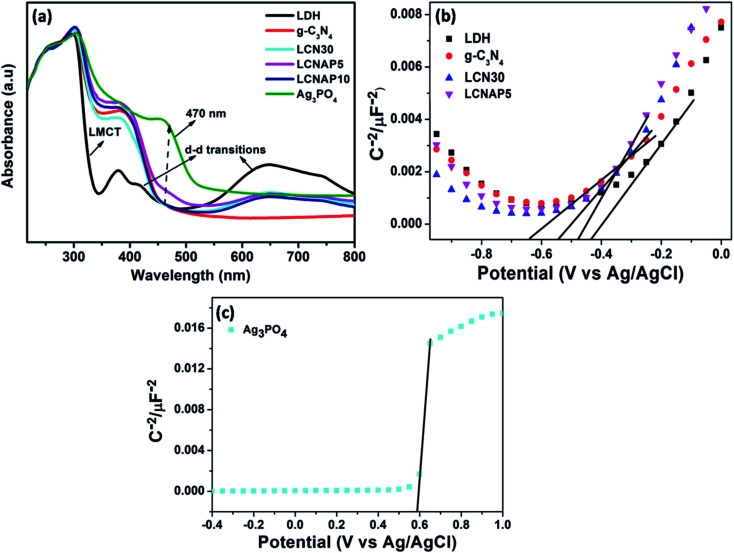
(a) UV-DRS spectra and (b and c) M–S plots of pure NiAl-LDH, g-C_3_N_4_, Ag_3_PO_4_, and the LCN30 and LCNAP nanocomposites.

The water-splitting activity of a photocatalyst is particularly related to its optical absorption and band-structure characteristics. In this regard, UV-vis DRS and Mott–Schottky measurements were carried out to investigate the optical properties and energy band structures of NiAl-LDH, Ag_3_PO_4_, LCN30, and LCNAP photocatalysts, as presented in Fig. 7. NiAl-LDH displays three apparent absorption bands; the one that appears in the UV range (200 to 300 nm) is assigned to ligand–metal charge transfer (LMCT) from a transition occurring between the 2p orbital of O and the 3d orbitals of Ni^2+^ ions in the LDH layer, and the other two broad absorption bands located in the visible range (300–500 nm and 600–800 nm) correspond to the d–d transitions of Ni^2+^ ions with MO_6_ octahedral geometry (Fig. 7a). The ^3^A_2g_ (F) → ^3^T_1g_ (P) and ^3^A_2g_ (F) → ^3^T_1g_ (F) spin-allowed transitions give rise to bands at 380 and 740 nm and the bands at 420 and 640 nm correspond to the spin-forbidden transitions of ^3^A_2g_ (F) → ^1^T_2g_ (D) and ^3^A_2g_ (F) → ^3^T_2g_ (D), respectively.^37,53,54^ Furthermore, the light absorption of NiAl-LDH is enhanced in the visible range upon the addition of g-C_3_N_4_, which indicates strong co-ordination between NiAl-LDH and g-C_3_N_4_ in the resulting LCN30 heterojunction. Upon the loading of Ag_3_PO_4_, the LCNAP composites possess significantly improved absorption in the visible region (470 nm) compared to the other two samples (NiAl-LDH and LCN30) and there is an obvious red shift of the absorption edge. According to the UV-DRS spectra, the bandgap energies estimated from Tauc plots of NiAl-LDH, g-C_3_N_4_, Ag_3_PO_4_, LCN30, and LCNAP5 are 2.53, 2.69, 2.26, 2.62, and 2.56 eV, respectively (Fig. S3†). Furthermore, it was observed that the bandgap of LCNAP5 was considerably decreased after the loading of Ag_3_PO_4_. In addition to a suitable bandgap, determining the position of the valence band and the conduction band of a prepared photocatalyst is also extremely important when constructing a photocatalyst. As shown in Fig. 7b and c, M–S plots of pure NiAl-LDH, g-C_3_N_4_, Ag_3_PO_4,_ and composite LCN30 and LCNAP5 samples were obtained. All samples showed positive slopes in the M–S plots, suggesting that they are n-type semiconductors. Furthermore, the flat-band potential (*V*_fb_) can be determined based on the Mott–Schottky (M–S) plots. The estimated flat-band potentials are −0.43, −0.64, 0.58, −0.54, and −0.47 V *vs.* Ag/AgCl (at pH = 7) for pure NiAl-LDH, g-C_3_N_4_, Ag_3_PO_4_, and the LCN30 and LCNAP5 composites, respectively. Moreover, the carrier densities (*N*_D_) can be estimated based on the slopes of the Mott–Schottky plots. The calculated carrier density values of pure NiAl-LDH, g-C_3_N_4_, Ag_3_PO_4_, and the LCN30 and LCNAP5 nanocomposites are reported in Table 1. They show that after the formation of a composite between LCN30 and Ag_3_PO_4_, the carrier density was significantly increased compared with pure samples. Therefore, the LCNAP5 composite can improve charge separation and extend the photoresponse of the photocatalyst, resulting in improved photocatalytic water splitting activity. This suggests the creation of superior performance upon the deposition of g-C_3_N_4_ and Ag_3_PO_4_.

**Table 1:** Flat-band potentials and carrier concentrations (*N*_D_) of the studied materials

Catalyst	Flat-band potential (*V*_fb_; V)	Carrier concentration (*N*_D_; cm^−3^)
NiAl LDH	−0.43	3.88 ×10^21^
g-C_3_N_4_	−0.64	6.25 × 10^21^
Ag_3_PO_4_	0.58	1.83 ×10^21^
LCN30	−0.54	7.30 × 10^21^
LCNAP5	−0.47	9.39 ×10^21^

### 3.4. Wettability and water contact angles

**Fig. 8:**
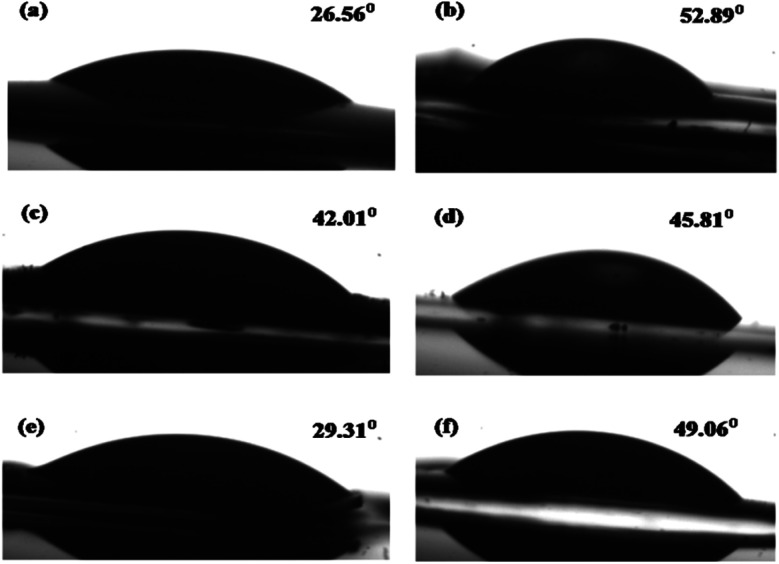
The water contact angles of (a) NiAl-LDH, (b) g-C_3_N_4_, (c) LCN30, (d) Ag_3_PO_4_, and the (e) LCNAP5 and (f) LCNAP10 nanocomposites.

The surface wettability is intimately related to the catalytic performance, and it involves the interaction between a liquid and solid in contact. Water contact angles were measured to verify the wetting behaviors of the prepared samples. As shown in Fig. 8a and b, pure NiAl-LDH, gCN, and the LCN30 nanocomposite exhibited contact angles of 26.56°, 52.89°, and 42.01°, respectively.^55,56^ Moreover, improved hydrophilicity was achieved upon the further modification of LCN30 with Ag_3_PO_4_, and the contact angles were found to be 29.31° and 49.06° for LCNAP5 and LCNAP10, respectively (Fig. 8e and f). LCNAP5 is significantly more hydrophilic than LCNAP10 (as indicated by the higher water contact angle value). The water contact angle of a photocatalyst is different when the surface functional groups are different. The excess amount of Ag_3_PO_4_ controls the water contact angle of LCNAP10 because it blocks the OH groups of the photocatalyst surface. It is known that when surfaces have hydrophilic properties, the photocatalytic performance is also high.

### 3.5. Photocatalytic overall water splitting activities

**Fig. 9:**
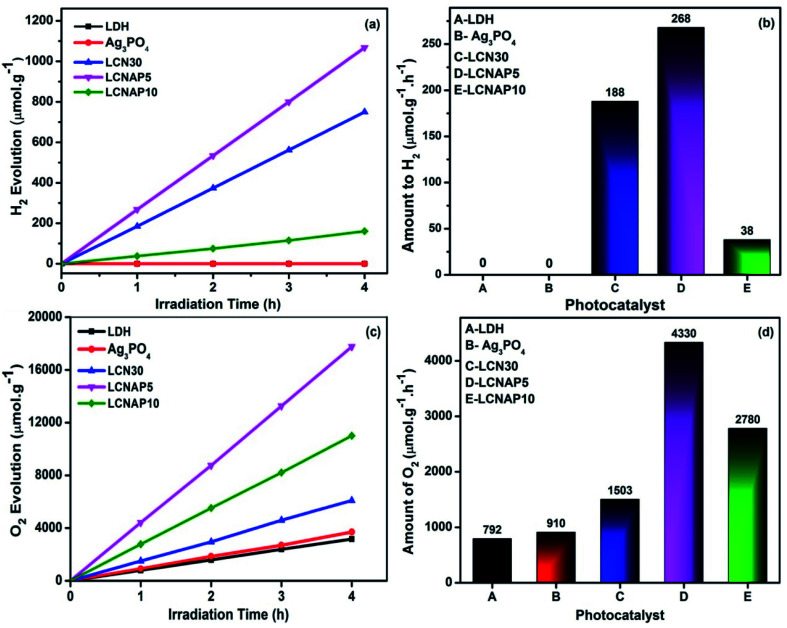
The time depended and comparative plots of pure NiAl LDH, Ag_3_PO_4_ and LCN30, LCNAP nanocomposites (a and b) water reduction and (c and d) water oxidation.

**Fig. 10:**
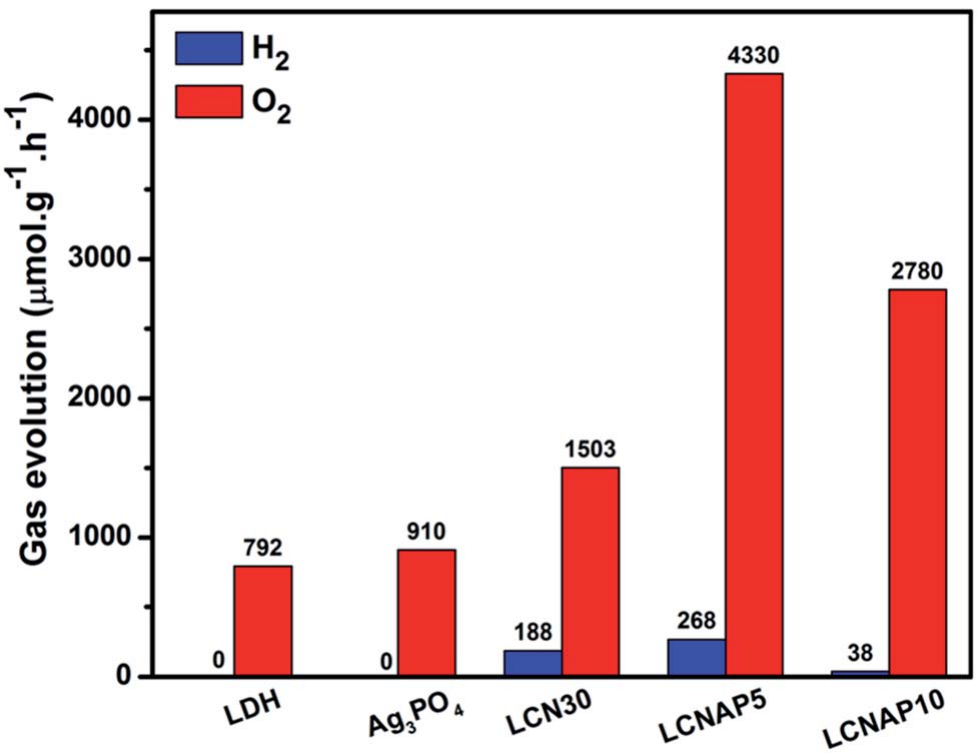
The photocatalytic overall water splitting performances of the prepared samples under light irradiation.

Fig. 9 shows photocatalytic H_2_ evolution from aqueous methanol solution and O_2_ evolution from aqueous silver nitrate. The length of H_2_ and O_2_ evolution using pure NiAl-LDH, Ag_3_PO_4_, and the LCN30 and LCNAP nanocomposites was 4 h, and a 250 Watt QTH lamp was used (Fig. 9a and c). Fig. 9b and d shows that only the composite samples (LCN30 and LCNAP) can evolve H_2_ and O_2_, whereas pure NiAl-LDH and Ag_3_PO_4_ could not produce H_2_ gas. Further, the overall water splitting activity was improved upon the addition of Ag_3_PO_4_. When forming a ternary composite with the ratio seen in LCNAP5, higher rates of gas evolution were obtained [H_2_ = 268 μmol g^−1^ h^−1^; O_2_ = 4330 μmol g^−1^ h^−1^] compared with the LCN30 composite. The apparent quantum yields with the LCNAP5 nanocomposite are 1.29% (H_2_) and 41.71% (O_2_). Comparisons of H_2_ and O_2_ evolution performance between relevant reported photocatalytic systems and the LCNAP5 nanocomposite are presented in Tables 2 and 3. The gas evolution rates of pure and composite samples are shown in Fig. 10; the gas evolution performance dropped when 10 wt% Ag_3_PO_4_ was used. This concentration dependence is attributed to the overloading of Ag_3_PO_4_ on the LCN30 surface. Unfortunately, the large amount of Ag_3_PO_4_ that seems to occupy the active centers on the surface of LCN30 may hinder light penetration into the composite material, and it might restrict interfacial charge transfer between NiAl-LDH, g-C_3_N_4_, and Ag_3_PO_4_, leading to lower H_2_ and O_2_ production with the LCNAP10 nanocomposite. The NiAl-LDH/g-C_3_N_4_/Ag_3_PO_4_ composite with a loading amount of 5 wt% Ag_3_PO_4_ revealed the best photocatalytic water reduction and oxidization efficiencies.

**Table 2:** A comparison of the H_2_ evolution performances of relevant photocatalysts

Photocatalyst	Light source	Sacrificial donor	H_2_ production, μmol g^−1^ h^−1^	Reference
NiAl-LDH/gC_3_N_4_/Ag_3_PO_4_	250 W quartz tungsten halogen lamp	Methanol	268	This work
Co(OH)_2_/gC_3_N_4_	300 W Xe lamp	TEOA	0	57
Co_3_O_4_/gC_3_N_4_	Visible light	TEOA	50	58
Pt/NiTi-LDH	300 W Xe lamp	Lactic acid	153	59
Pt/MgAlTi-LDH	300 W Xe lamp	Lactic acid	49	59
Au/layered H_2_SrTa_2_O_7_	300 W Xe lamp	—	17.5	60
NYF@Ag_3_PO_4_@black phosphorus	Laser light	Glycerol	145.9	61
Ag_3_PO_4_/polyoxometalates/GO	500 W Xe arc lamp	Methanol	50.75	62
Co_2_P/gC_3_N_4_	300 W Xe lamp	TEOA	53.3	63

**Table 3:** A comparison of the O_2_ evolution performances of relevant photocatalysts

Photocatalyst	Light source	Sacrificial donor	O_2_ production, μmol g^−1^ h^−1^	Reference
NiAl-LDH/gC_3_N_4_/Ag_3_PO_4_	250 W quartz tungsten halogen lamp	AgNO_3_	4330	This work
ZnCr-LDH/layered TiO_2_	450 W Xe lamp	AgNO_3_	1180	64
ZnCr-LDH/RGO	450 W Xe lamp	AgNO_3_	1200	65
gC_3_N_4_/NiFe-LDH	125 W mercury lamp	AgNO_3_	886	66
NiFe-LDH/N-rGO/g-C_3_N_4_	125 W Hg lamp	AgNO_3_	1280	7
Ag@Ag_3_VO_4_/ZnCr-LDH	150 W Hg lamp	AgNO_3_	571	67
TbZnCr-LDH	150 W Xe lamp	AgNO_3_	1022	68
MoSe_2_/Ag_3_PO_4_	25 mW white light LED	AgNO_3_	182	69
TiO_2_@CoAl-LDH	300 W Xe lamp	AgNO_3_	2240	70
Ag_3_PO_4_/graphdiyne/g-C_3_N_4_	300 W Xe lamp	AgNO_3_	753.1	71

**Fig. 11:**
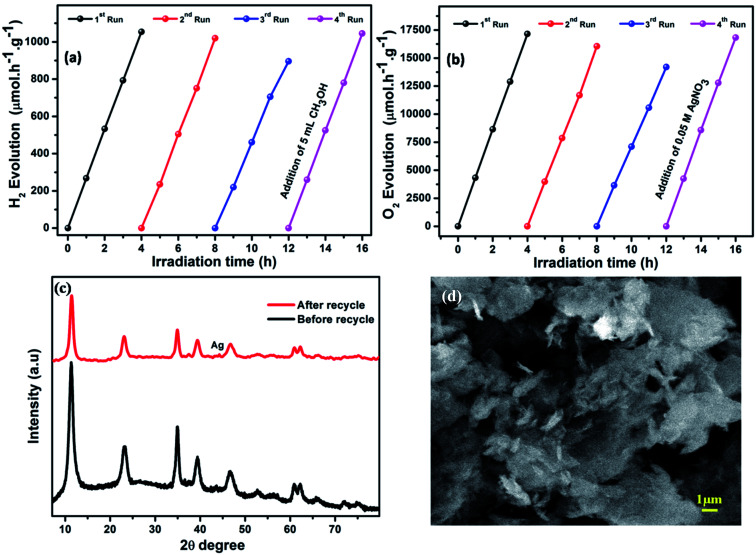
Cycling study of LCNAP5 during light-driven (a) water reduction and (b) water oxidation reactions. (c) XRD patterns of LCNAP5 before and after 4 cycles of water oxidation. (d) An SEM image of LCNAP5 after 4 cycles of water oxidation.

The photocatalytic recyclability and stability of a photocatalyst are very important for its practical application. Therefore, cyclic testing of LCNAP5 was carried out to estimate the H_2_ and O_2_ production stability of the catalyst (Fig. 11a and b) under light irradiation for 4 days, with each experiment conducted for 4 h. After the end of the third cycle, the catalytic activity was slightly decreased, although the water reducing activity of LCNAP5 remained at 84% (Fig. 11a). At the same time, the water oxidation activity of LCNAP5 was maintained at 82% (Fig. 11b). Thereafter, upon the addition of the sacrificial reagents methanol (CH_3_OH) and silver nitrate (AgNO_3_) to the same reaction mixture, the photocatalyst almost regained its maximum activity. Hence it is clear that a decreased concentration of sacrificial reagents was the main reason for the declining activity. This confirmed that the LCNAP5 nanocomposite is stable for both H_2_ and O_2_ production during long-term photocatalytic reactions.

As shown in Fig. 11c and d, X-ray diffraction patterns and SEM images of the LCNAP5 nanocomposite after 4 cycles of water oxidation were obtained. One new peak located at 44.2° was seen in the XRD pattern after the photocatalytic reaction, which indicates that some Ag_3_PO_4_ has decomposed to Ag.^69^ Ag could cover active sites on the surface of LCNAP5, resulting in a decrease in the photocatalytic efficiency. However, only a small amount of Ag was formed during the recycling process, so no obvious differences were observed in the SEM image after 4 cycles of water oxidation.

### 3.6. Photoelectrochemical and photoluminescence analysis

**Fig. 12:**
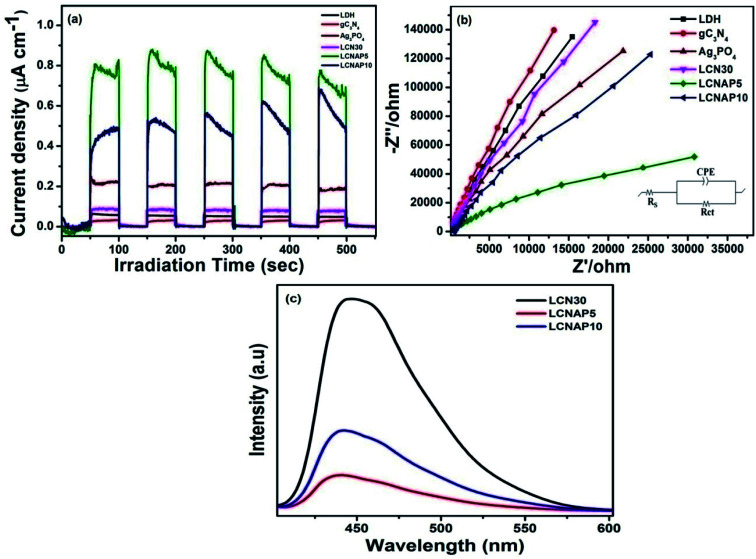
(a) Photocurrent responses and (b) Nyquist impedance plots of pure and composite samples. (c) PL emission spectra of LCN30, LCNAP5, and LCNAP10 photocatalysts.

The charge-carrier generation behavior and their migration abilities in the LCNAP ternary composite were verified based on the transient photocurrent response and electrochemical impedance spectroscopy (EIS) under light irradiation. As shown in Fig. 12a, the photocurrent density of LCNAP5 is much higher than those of NiAl-LDH, g-C_3_N_4_, Ag_3_PO_4_, LCN30, and LCNAP10, suggesting efficient photogenerated electron–hole-pair separation. A higher photocurrent intensity suggests a slower recombination rate of photoinduced carriers. Furthermore, the charge-carrier lifetime can be obtained from the photocurrent response using the following kinetic equation:^47,72,73^
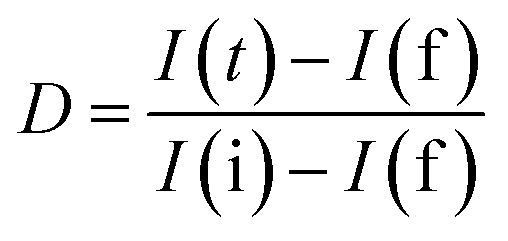
where *I*(*t*) denotes the current at a certain time, and *I* is the photoanode current where ‘i’ and ‘f’ represent the initial and final states, respectively. It is found that the lifetimes of NiAl-LDH, g-C_3_N_4_, Ag_3_PO_4_, LCN30, LCNAP5, and LCNAP10 are 5.10, 4.21, 6.89, 6.39, 11.65, and 7.40 s, respectively. The carrier lifetime of 5 wt% LCNAP indicates a decrease in the recombination rate and an increase in the lifetime of photogenerated excitons. To further verify the efficient charge transfer and electrical conductivity, EIS Nyquist plot measurements were carried out, and the results are shown in Fig. 12b. The inset shows the equivalent circuit model, in which *R*_s_, *R*_ct_, and CPE are the internal resistance, charge-transfer resistance, and a parallel constant phase element, respectively. The calculated *R*_ct_ values for NiAl-LDH, g-C_3_N_4_, Ag_3_PO_4_, LCN30, LCNAP5, and LCNAP10 are 70.67, 92.5, 40.27, 53.02, 18.03, and 33.76 kΩ,^74^ respectively. As LCNAP5 has lower charge transfer resistance, it revealed enhanced capacitance behavior and less resistance against charge-carrier diffusion. Generally, a small radius indicates high charge-carrier transfer. Compared with LCN30, the impedance spectrum of the LCNAP5 composite shows a smaller diameter, indicating greatly enhanced charge-carrier transfer due to the superior electrical properties of Ag_3_PO_4._

Subsequently, the photoluminescence (PL) emission was studied to determine the photogenerated electron and hole separation efficiencies of the photocatalysts. Fig. 12c shows the PL spectra of LCN30, LCNAP5, and LCNAP10 using an excitation wavelength of 380 nm. LCNAP5 exhibited a lowered recombination rate of excitons in comparison to LCNAP10, which might be due to the excess loading of Ag_3_PO_4_ on the surface of LCN30. The results obtained from the PL measurements of LCNAP5 support the photocurrent measurement results, which indicated that electron–hole-pair separation was improved upon introducing Ag_3_PO_4_ on LCN30, which further leads to efficient photocatalytic evolution.

### 3.7. Mechanism of the dual direct Z-scheme system

**Fig. 13:**
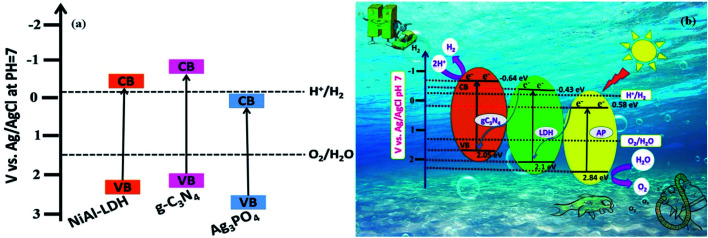
(a) The band structures of the pure samples. (b) A schematic diagram of the photocatalytic water splitting mechanism over the LCNAP photocatalyst system under light irradiation.

The band alignment between NiAl-LDH, g-C_3_N_4_, and Ag_3_PO_4_ leads to the formation of a Z-scheme system, which plays a crucial role in determining the photocatalytic water splitting activity of the NiAl-LDH/g-C_3_N_4_/Ag_3_PO_4_ nanocomposite. According to the M–S plots, the estimated *E*_CB_ values of NiAl-LDH, g-C_3_N_4_, and Ag_3_PO_4_ were −0.43, −0.64, and 0.58 eV, respectively.^75^ Moreover, the VB position can be determined using the following relation:^31^*E*_CB_ = *E*_VB_ − *E*_g_where *E*_CB_ is the conduction band position and *E*_g_ is the bandgap. The calculated *E*_VB_ positions of pure NiAl-LDH, g-C_3_N_4_, and Ag_3_PO_4_ were 2.1, 2.05, and 2.84 eV, respectively. The conduction and valance band positions of NiAl-LDH, g-C_3_N_4_, and Ag_3_PO_4_ for photocatalytic water splitting are represented in Fig. 13a. The higher VB potential of Ag_3_PO_4_ exerts a strong driving force for the separation of photogenerated charge pairs in the space charge region. Based on the above results, the photocatalytic water splitting mechanism of NiAl-LDH/g-C_3_N_4_/Ag_3_PO_4_ is proposed, as shown in Fig. 13b. The coupling of semiconductors with various band structures can expand the visible light absorption range and enhance the lifetime of charge carriers due to charge separation. The charge transfer mechanism of the Z-scheme system is different from traditional and heterojunction photocatalysts; in this scheme, at least one material should satisfy either oxidation or reduction reaction conditions. Hence, electrons from the lower CB recombine with holes from the higher VB. Moreover, the electrons from the higher CB generate H_2_ and those from the lower VB generate O_2_. In this process, only half of the photoexcited electrons and holes are completely used. The introduction of Ag_3_PO_4_ into NiAl-LDH and g-C_3_N_4_ favors enhanced H_2_ and O_2_ evolution from the dual direct Z-scheme system. Under illumination, photoexcited electrons from the CB of Ag_3_PO_4_ can directly combine with holes from the VB of NiAl-LDH, while excited electrons migrate to the CB of NiAl-LDH. In the meantime, holes are left in the VB and electrons are stored in the CB of g-C_3_N_4_. The holes in the VB of Ag_3_PO_4_ and the electrons in the CB of g-C_3_N_4_ are used to improve the strong redox abilities for H_2_ and O_2_ evolution. In this process of photocatalytic water splitting, the evolution of hydrogen and the evolution of oxygen occur separately at opposite surfaces of the semiconductor photocatalyst due to the half-reaction system in the dual direct Z-scheme. Furthermore, the positive band potential of Ag_3_PO_4_ can be used as a hole carrier, providing effective charge transfer.

## 4. Conclusions

In summary, the effects of Ag_3_PO_4_ on the photocatalytic water splitting activity of NiAl-LDH/g-C_3_N_4_ sheets were investigated. The performance was much improved when using the LCNAP5 ternary nanocomposite compared to pure LDH and the LCN30 binary nanocomposite. It was established that LCNAP5 demonstrated enhanced water splitting activity due to promoted charge transfer and suppressed charge recombination. The results strongly suggest that during the water-splitting reaction both the water oxidation (O_2_ evolution) and reduction (H_2_ evolution) reactions are enhanced after the addition of Ag_3_PO_4_. This work not only develops a NiAl-LDH/g-C_3_N_4_/Ag_3_PO_4_ ternary nanocomposite with high photocatalytic activity to achieve overall water splitting but it also provides deeper understanding for constructing efficient dual direct Z-scheme systems.

## Conflicts of interest

There are no conflicts to declare.

## Supplementary Material

NA-003-D0NA01074J-s001
