# Supplementary material for: The construction of a dual direct Z-scheme NiAl LDH/g-C3N4/Ag3PO4 nanocomposite for enhanced photocatalytic oxygen and hydrogen evolution
Source: Nanoscale Adv. 2021 Feb 26;3(7):2075–88. doi: 10.1039/d0na01074j (PMC9418587; doi:10.1039/d0na01074j)
Supplement: NA-003-D0NA01074J-s001 [file NA-003-D0NA01074J-s001.pdf]

Electronic Supplementary Material (ESI) for Nanoscale Advances.
This journal is © The Royal Society of Chemistry 2021

Supporting Information

Construction of dual direct Z- scheme NiAl LDH/g-C_3N_4/Ag_3PO_4 nanocomposite for enhanced photocatalytic overall water splitting

*S. Megala[a], P. Ravi[b,c], P. Maadeswaran[d], M. Sathish[b,c], R. Ramesh[a]**

[a]Department of Physics, Periyar University, Salem-636011, Tamil Nadu, India

[b]Electrochemical Power Sources Division, Central Electrochemical Research Institute Karaikudi -630006, Tamil Nadu, India

[c]Academy of Scientific and Innovative Research (AcSIR), Ghaziabad-201002, India
[d]Department of Energy Science, Periyar University, Salem-636011, Tamil Nadu, India

*Corresponding author.

E-mail address:rameshphys@gmail.com

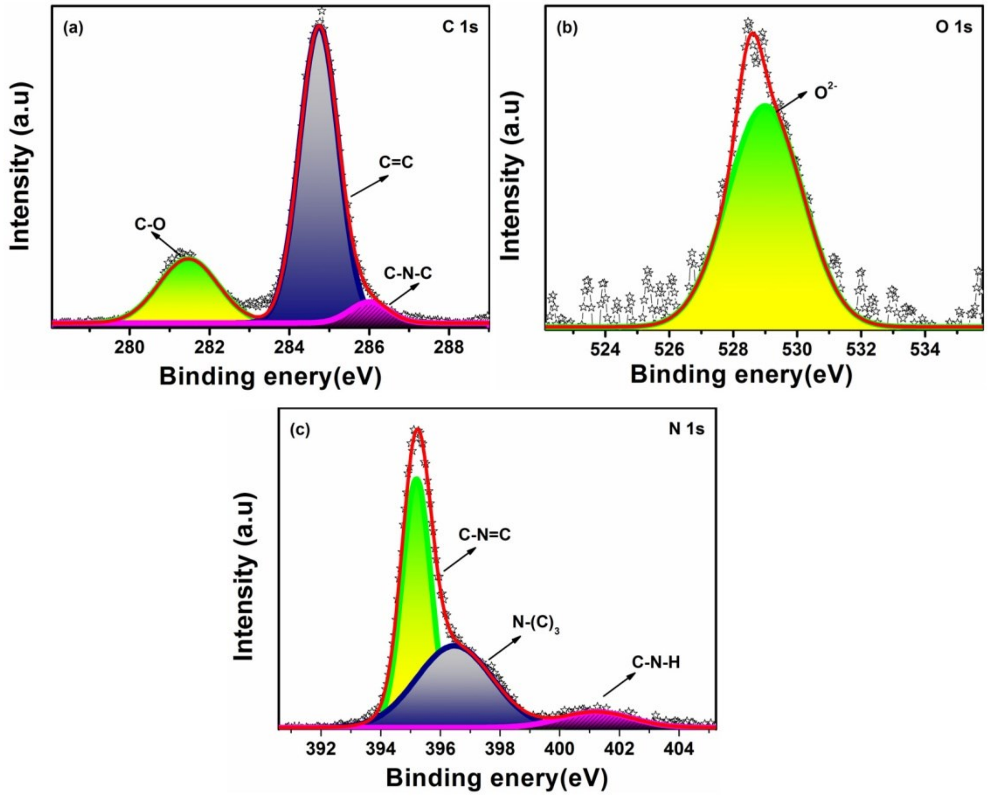

Fig. S1 High resolution XPS spectra of pure g-C_3N_4 for (a) C1s, (b) O 1s, and (c) N 1S.

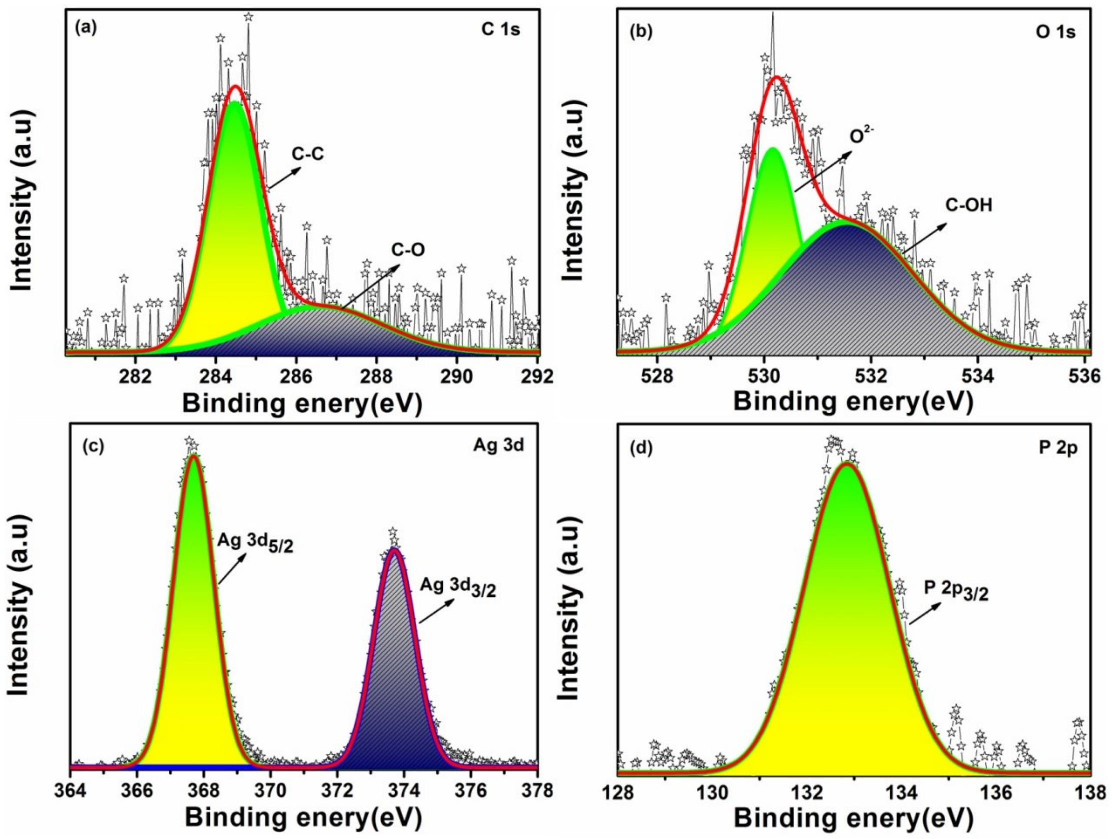

Fig. S2 High resolution XPS spectra of pure Ag_3PO_4 for (a) C1s, (b) O 1s, (c) Ag 3d, and (d) P 2p.

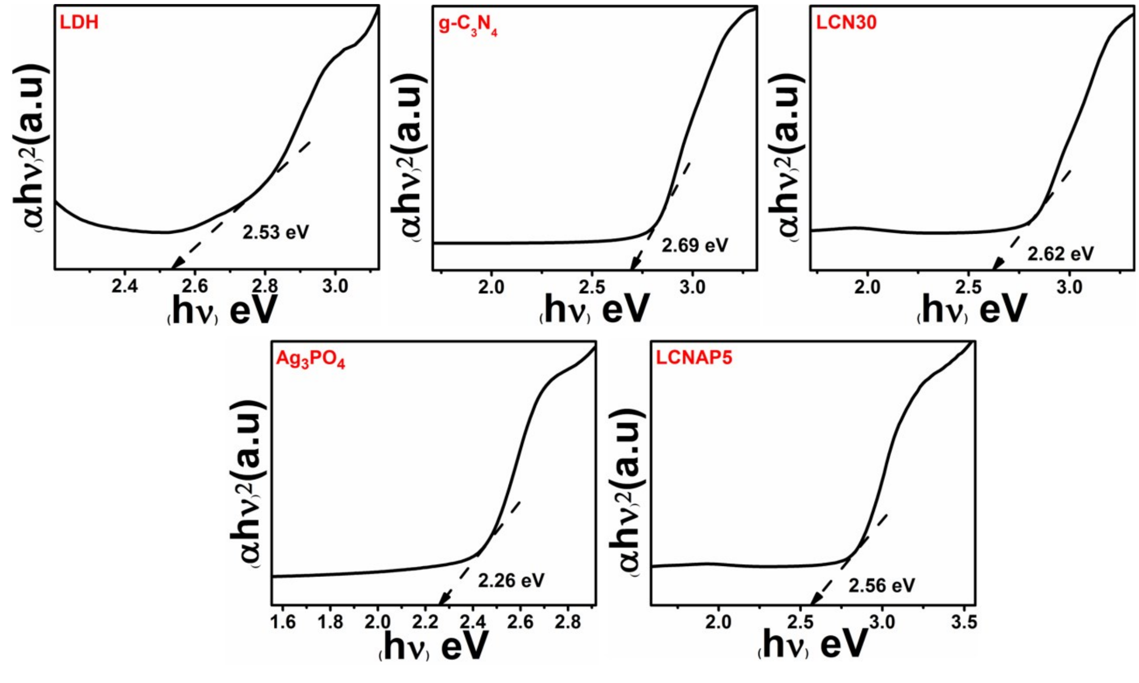

Fig. S3 Tau plots of NiAl LDH, g-C_3N_4, Ag_3PO_4 and LCN30, LCNAP nanocomposites.
